# Young people's and adults' views and experiences of decision‐making to manage compromised first permanent molars: a qualitative study

**DOI:** 10.1111/ipd.13217

**Published:** 2024-05-27

**Authors:** Greig D. Taylor, Catherine Exley, Nicola Innes, Christopher Robert Vernazza

**Affiliations:** ^1^ School of Dental Sciences Newcastle University Newcastle UK; ^2^ Population Health Sciences Institute Newcastle University Newcastle UK; ^3^ School of Dentistry Cardiff University Cardiff UK

**Keywords:** molar, qualitative, shared decision‐making

## Abstract

**Background:**

Little information on young people's and adults' views and experiences on decision‐making for managing compromised first permanent molars (cFPM) exists.

**Aim:**

To establish young people's and adults' views and experiences of decision‐making for managing cFPM.

**Design:**

Face‐to‐face (online) semi‐structured interviews were undertaken using an iteratively designed topic guide. Participants aged 12–65 were purposively sampled with recruitment from different dental clinics (three primary care, an out‐of‐hours emergency and one dental hospital). Interviews were audio‐recorded, transcribed verbatim and analysed using thematic analysis.

**Results:**

Three themes were generated from young people's interviews (*n* = 9): (i) influencing factors; (ii) long‐term considerations; and (iii) shared decision‐making. Three themes were generated from adults' interviews (*n* = 13): (i) influences that affect decisions; (ii) perceptions of the specialist's role; and (iii) importance of shared decision‐making for children and young people.

**Conclusion:**

Several factors influenced decision‐making; for young people, professional opinions were important, and parental/peer influences less so. For adults, it was based on decisions on their prior experiences. Adults felt young people were abnormal if referred to a specialist. Young people wanted autonomy in decision‐making to be respected; in reality, their views were rarely heard. There is potential to increase young people's involvement in shared decision‐making for cFPM, which aligns with their aspirations.

**Why this paper is important to paediatric dentists**

- Exploration of factors and influences should be included when discussing how best to manage cFPM.
- Young people should be actively involved in as part of a shared decision‐making dynamic for cFPM to ensure their autonomy is being respected.
- Professionals need to appreciate the role they have in any shared decision‐making process.

## 1 INTRODUCTION

Determining optimum management strategies for a compromised first permanent molar (cFPM) is not well‐understood. Whilst there is no agreed consensus, a working definition of a cFPM, used in this research and manuscript, is a restorable first permanent molar with either distinct cavitation (ICDAS Codes 5 & 6)^1^ due to dental caries, or post‐eruptive breakdown due to molar–incisor hypomineralisation.^2^ Management options for cFPM are varied, as evidenced by previous quantitative studies^2^, ^3^, ^4^ but can be simply categorised into restoration or extraction; there is, however, insufficient evidence for the factors that influence the outcomes of the different options. This makes explaining treatment options difficult for clinicians and compromises overall decision‐making.^3^ Patients might select a treatment based on how well they understand the options presented to them. This decision, however, may also be influenced by their underlying preferences and values.^5^ Acknowledging preferences is a vital component of shared decision‐making as it helps clinicians come to an agreement with patients/parents on the appropriate treatment option. Such preferences and values can be hard to elicit, especially in children and young people who may not be afforded the opportunities to disclose them.^6^

Deciding how to manage cFPM should be a shared process, which involves the child, their parent/guardian and the clinician.^5^ Children and young people should be involved in any decision‐making process that directly impacts their health,^5^ but in reality, it can be quite complex to do so.^7^ They might not have the competence and capacity to understand the implications of the decisions they are making.^7^ Given that management options for cFPM are varied and have lifelong implications, deciding how best to manage cFPM has to be tailored on an individual basis.

There is a paucity of evidence on how young people, parents and adults make decisions for cFPM and establishing their views is critical, to increase children's autonomy and independent decision‐making for choices that affect their lives.^8^ Therefore, the aim of this study was to establish young people's and adults' views and experiences of decision‐making to manage cFPMs.

## 2 MATERIALS AND METHODS

A qualitative methodology was used for this study. Favourable ethical opinion was obtained from the North of Scotland ethic service (20/NS/0124; 22 October 2020). Appropriate informed consent and assent of participants were obtained. Reporting in this article is in line with consolidated criteria for reporting qualitative research recommendations, and the checklist is included in Appendix S1.^9^

### 2.1 Sample

A purposive sampling strategy was used to recruit participants with variation within specified characteristics that might influence their perceptions (Table 1). The characteristics were specifically chosen as the early qualitative findings from this study were to be used to inform the design of a subsequent young person and public preference elicitation study.^10^, ^11^ The sample included two separate cohorts: young people (12–16 years old) and (non‐related) adults.

**TABLE 1:** Patient characteristics used during purposive sampling.

Characteristics	Variation within characteristics
Age	Young person interviewee: 12–16 years old Adult interviewee: 17–65 years old
Gender	As described by the participant
Oral health experiences	Those who access and do not access dental care regularly, as described by the participant
Oral health treatment experiences	Those who have had the experience of a restoration, an extraction, both and no treatment on first permanent molar
Parent	Those who are parents and those who are not

### 2.2 Recruitment

Participants were recruited from three primary care general dental clinics, an out‐of‐hours emergency clinic and one tertiary dental hospital based in North East England. Participants were approached by their treating clinicians and given information about the study. Eligibility was confirmed, and their desired characteristics (Table 1) were recorded. If interested, the desired characteristics and contact information were sent, via an encrypted secure email, to the lead author (GT). Potential participants were contacted a few days later to confirm their participation. Recruitment was targeted to ensure that sufficient representation of the desired characteristics was included across the sample. If a certain characteristic was underrepresented in early participants, future participants with that desired characteristic were targeted for recruitment. Recruitment ended when data saturation was reached, as determined by no new information being generated in the interviews.^12^

### 2.3 Interviews

One‐to‐one (online) semi‐structured interviews were conducted by one researcher (GT). GT is a male clinical academic in paediatric dentistry who has been formally trained in qualitative methods and has experience in conducting qualitative studies. GT regularly provides dental care as a specialist paediatric dentist, and emergency dental care to both young people and adults. In the interviews, GT presented himself as a researcher to participants but disclosed his profession if asked. The purpose of removing focus from the clinical role was to allow participants to talk without being distracted by the knowledge they were talking to a clinician, which may have impacted the information they shared or turned the interview into a clinical consultation.^13^ Young people were interviewed on their own or with an adult present if the young person preferred this. If an adult was present, the conversation was directed to the young person.

A topic guide, developed using the scientific literature and through discussion with the research team, comprised the following:

- Section 1—dental attendance, general dental experiences and expectations;
- Section 2—identification of the first permanent molar and exploring experiences specifically with these teeth;
- Section 3—how decisions were made for their cFPM, with slight variations for those who have received treatment for their cFPM and those who had not; and
- Section 4 (for adult interviews only)—how to manage a cFPM in their child or a hypothetical child if they were not parents.

The topic guide was iteratively adapted, following a constant comparative approach, to allow further exploration of ideas highlighted in earlier interviews.^14^

**FIGURE 1:**
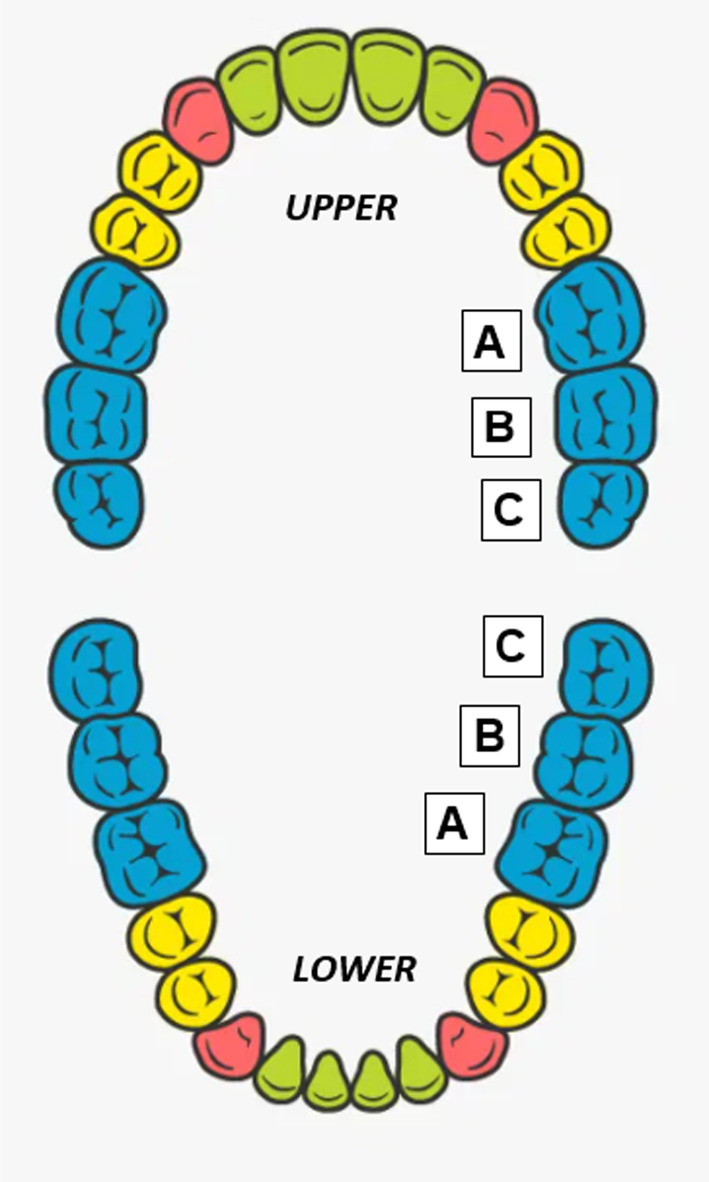
Identification of the first permanent molar for use in the interviews.

To ensure the interview focussed on the first permanent molar, participants were asked to identify this tooth using a staged questioning approach using a diagram to supplement this process (Figure 1). If incorrectly identified, participants were informed of the correct tooth. It was emphasised that all future questions and discussions were about this tooth. The diagram remained on the screen throughout the interview.

### 2.4 Piloting

Two pilot interviews (one with an adult and one with a young person) were conducted with one volunteer familiar to the interviewer and the other unfamiliar, to test the topic guide and diagram sharing (Figure 1). This permitted re‐organisation of the guide to improve flow. These pilot interviews were not analysed.

### 2.5 Data handling/analysis

Interviews were audio‐recorded, transcribed verbatim (using an online transcription company) and anonymised. Field notes were completed to support these recordings. Transcribed data were entered into NVivo version 12©^15^ and checked for accuracy by re‐reading whilst listening to the sound wave file.

A reflexive thematic analysis^16^ was used to analyse the data, adopting a constant comparative approach.^14^ Two adult and young person manuscripts were initially analysed by GT to label interesting sections of dialogue. These pieces of data (initial codes) were discussed with CE, an experienced qualitative researcher, to mitigate against superficial coding of data and aid reflexivity.^17^ Reflexivity was considered by all members of the research team during the analysis process.^13^, ^17^ Consideration and caution were applied to consider how members' personal opinions and experience might influence analysis. Subsequent analysis was completed by GT, enabling themes to be searched and reviewed in later transcripts. Regular research team meetings (GT, CE, NI and CRV) supported the iterative process of developing themes. Initial themes identified during earlier analyses were re‐analysed and triangulated, using field notes and observations in later interviews, to ensure their validity. Subsequent analysis was completed alongside continual review of the data sets, by GT, to ensure that no potentially significant information was overlooked. This process continued until interviewing generated no additional new information,^12^ at which point these data were considered saturated. Finally, themes were refined during a whole team discussion. Participants were offered the opportunity to clarify their transcripts and provide feedback, should they wish to do so.

## 3 RESULTS

### 3.1 Adolescent interviews

Nine young people were interviewed (Table 2). Interview lengths ranged from 22 min 14 s to 39 min 9 s, with an average interview length of 34 min and 5 s. Two young people completed the interview in the presence of their parent. Eight of the nine young people identified the cFPM correctly and independently as shown in Figure 1.

**TABLE 2:** Characteristics of participating young people (*n* = 9).

Participant	Age	Gender	Oral health experiences	Treatment experience
Adolescent 1	13	Male	Regular	Nil
Adolescent 2	12	Female	Regular	Nil
Adolescent 3	16	Male	Regular	Fill and extract
Adolescent 4	12	Male	Regular	Fill
Adolescent 5	13	Male	Irregular	Extract
Adolescent 6	15	Female	Regular	Nil
Adolescent 7	14	Female	Regular	Fill
Adolescent 8	14	Male	Regular	Nil
Adolescent 9	12	Female	Regular	Fill and extract

Three major themes, and sub‐themes, were generated (Table 3). Each quotation included the respondent ID and relevant characteristics to inform a sub‐group ID number (age, gender, dental attendance and extraction/filling experience), for example, Adolescent 6 (15, F, Regular, Nil) of who provided the quote.

**TABLE 3:** Themes (and sub‐themes) of adolescent interviews (*n* = 9).

Final themes (and sub‐themes)		
Influential decision‐making factors	Long‐term considerations	Shared decision‐making
Professional opinion External—peers and parents Personal prior experiences Acquired condition vs. Developmental	Preference for tooth retention Recurrent pain leads to extraction	Provision of information Asserting autonomy Trust professional opinion

### 3.2 Theme 1: Influential decision‐making factors

The opinion of the dentist was a key influencing young people's decisions on how they want to manage cFPM:

I'd want my dentist's advice, ask them which one they thought was better, and then probably make a decision off that… Adolescent 6 (15, F, Regular, Nil)

The views of clinicians were often more important/valuable than those of others:

They [dentists] know more than your parents about teeth, so I'd listen more to what they suggested about how to treat them… Adolescent 2 (12, F, Regular, Nil)

Young people of shaped their decisions by drawing on oral health philosophies/beliefs instilled in them by their parents:

…my parents have always taught me to like keep my teeth, just like they have… Adolescent 4 (2, M, Regular, Fill)

I've had an extraction and a filling of this back molar as my parents have had both before, so they must be ok options… Adolescent 3 (16, M, Regular, Fill/Extract)

Young people additionally also spoke about how they would draw on prior treatment experiences to inform decisions for cFPM, now or in future:

Having a tooth yanked was canny [very/really] sore, but I'd still have it done again if that's what I chose to have done… Adolescent 5 (13, M, Irregular, Extract)

Whilst a clinician's opinion and values instilled in them by their parents were important in decision‐making, it seems that young people's peers were less likely to influence their decision‐making. Conversations occurred with friends about their own treatment experiences but had little direct influence on decision‐making:

I'd ask my friends what they [have] had done. I'm sure if I chose a filling, they wouldn't be fussed or mean to me about it, if they had an extraction, as if they did, then they're not your friends, are they? Adolescent 1 (13, M, Regular, Nil)

The physical size of the defect appeared to influence decision‐making. It was assumed the larger the defect, the more complex the work to save it would be, so removal may be best, although there were some discrepancies about what ‘big’ meant.

…if it's bigger then it will take a bit more time to fill and could hurt more, so take it out, as if it was a bit bigger then it would be harder to treat… Adolescent 8 (14, M, Regular, Nil)

It just depends on how like really big it is, but what I mean by big I do not really know… Adolescent 4 (12, M, Regular, Fill)

The cause of the cFPM affected young people's decisions. If the cFPM was due to a developmental condition, rather than an acquired condition, there was more inclination towards restoration:

I'd not be as worried because I hadn't caused it, but this would make me want to try and save it, as I was born with it… Adolescent 2 (12, F, Regular, Nil)

This was, however, not consistently felt to be the case:

…it doesn't really matter whether I caused this, or [if] I was born with it, if the hole is too big, I'd have it extracted. Adolescent 5 (13, M, Irregular, Extract)

…if it did not develop and was going to form in a different way then it is not really worth trying to salvage the tooth with that. I would say get rid of it. Adolescent 6 (15, F, Regular, Nil)

### 3.3 Theme 2: Long‐term considerations

Young people preferred to retain the cFPM where possible, taking into consideration the long‐term implications of this decision:

When deciding to choose between filling or extraction, and how it would impact me as an adult, I would prefer to keep hold of the tooth and retain it… Adolescent 8 (14, M, Regular, Nil)

This initial preference for restoration was trivial if the tooth was to become sore later in life. The presence of pain would likely prompt a change towards deciding to extract this tooth and overrode the option of endodontic treatment:

…if it was a tooth with a filling that was painful when I chewed. So, if that would kind of not go away with another filling in, then I would definitely want an extraction… Adolescent 3 (16, M, Regular, Fill/Extract)

…I think if the root canal treatment meant having to keep up regular maintenance of the tooth, a lot of times over kind of multiple years, then I would say it's not worth it. Just get the extraction. Adolescent 9 (12, F, Regular, Fill/Extract)

### 3.4 Theme 3: Shared decision‐making

It was clear that young people want to assert and express their autonomy when making healthcare decisions:

…what happens with my teeth is my choice, but how often I get to make that choice I am not sure… Adolescent 2 (12, F, Regular, Nil)

…I felt like I had a choice in the matter, rather than my parents telling me what to do, which is something that is really important to me… Adolescent 3 (16, M, Regular, Fill/Extract)

Young people want professionals to give them information, to support their decision‐making, but retain the desire to make their autonomous decisions:

I think it's important to have information focussing on what would happen and what could happen in the future to help you make your decision… Adolescent 9 (12, F, Regular, Fill/Extract)

I think lay it all out – it's always good to lay out all of the options. It would be less useful if they [dentist] just said this is definitely the wrong option, this is definitely the right option. Adolescent 6 (15, F, Regular, Nil)

### 3.5 Adult interviews

Thirteen adults were interviewed (Table 4) to establish their opinions of managing their own cFPM and how they would make decisions for their own child, or a hypothetical child if they were not a parent. Of the 13 adults, eight had children and five did not. Interviews ranged in duration from 27 min 36 s to 43 min 17 s, with the average interview length being 36 min 47 s. Twelve of the 13 adults were able to identify the cFPM independently using Figure 1.

**TABLE 4:** Characteristics of adults (*n* = 13).

Participant	Age	Gender	Oral health experiences	Treatment experience	Parent
Adult 1	17	Male	Regular	Nil	No
Adult 2	25	Male	Regular	Fill	No
Adult 3	32	Female	Irregular	Extraction/Fill	No
Adult 4	20	Male	Irregular	Nil	No
Adult 5	43	Male	Regular	Extraction	Yes
Adult 6	37	Male	Regular	Fill	No
Adult 7	39	Female	Regular	Extraction	Yes
Adult 8	24	Female	Regular	Nil	No
Adult 9	48	Female	Regular	Extraction/Fill	Yes
Adult 10	53	Female	Regular	Fill	Yes
Adult 11	46	Male	Regular	Extraction/Fill	Yes
Adult 12	47	Male	Regular	Extraction/Fill	Yes
Adult 13	37	Female	Regular	Extraction/Fill	No

Three major themes, and sub‐themes, generated are shown in Table 5.

**TABLE 5:** Themes (and sub‐themes) of adolescent interviews (*n* = 13).

Final themes (and sub‐themes)		
Influences that affect decisions	Perception of specialist's role	Importance of shared decision‐making for cFPM in children and young people
Lived experiences Parental effect Societal constructs		Reality vs. abstract Empowering children

### 3.6 Theme 1: Influences that affect decisions

Adults rely heavily on previous lived experiences of a wide range of dental treatments, for example, filling, extraction, root canal treatment and when deciding how to manage cFPM in future:

I've had fillings and extractions in the past, extractions were a lot worse than the fillings, and that is going to influence what I decide to do in the future… Adult 3 (32, F, Irregular, Ext/Fill, Not‐Parent)

I've only experienced a filling, and it has worked, so I'm pretty content with having those done again… Adult 2 (25, M, Regular, Fill, Not‐Parent)

My previous experiences of root canal treatment prompted me to try it out again. However, I knew that if it did not work, like it did previously, then I was happy to get it [tooth] out. Adult 11 (46, M, Regular, Ext/Fill, Parent)

It appears adults' decisions are influenced by the behaviours and attitudes of their own parents. For some, their decisions mirrored their parents whilst for others, they did the opposite:

I'm always inclined to do whatever my parents did with their teeth… Adult 7 (39, F, Regular, Extraction, Parent)

I recall my parents mainly having teeth extracted, but I cannot say this fits with how I make decisions about my own teeth. Adult 9 (48, F, Regular, Ext/Fill, Parent)

In addition to own lived experiences and parental influence, ‘accepted’ societal constructs of the terms ‘filling’ and ‘extraction’ appear to influence decision‐making for adults:

Fillings seem like quite normal, so I do not think I would think of it as a big thing really…maybe that isn't the same for an extraction. You know they're [all} very different things in most people's eyes. Adult 12 (47, M, Regular, Ext/Fill, Parent)

Exploring this concept in more depth, it became apparent that procedural complexities associated with both a filling and an extraction underpinned the influence these constructs had on decision‐making:

I am sure it's well‐known that fillings are a little bit weird for about half an hour afterwards [as] you know, it's a fairly short‐lived experience and not difficult…whereas extractions are more invasive, takes longer and linked with more problems… Adult 9 (48, F, Regular, Ext/Fill, Parent)

### 3.7 Theme 2: Perceptions of specialist role

The role of the specialist in managing cFPM was perceived to be different from a general dentist's role. If specialist involvement was required, adults perceived the child's case to be more challenging:

Being sent to a specialist means it's more complex, so therefore that worries me a bit. Adult 13 (37, F, Regular, Ext/Fill, Non‐Parent)

A specialist referral, for some adults, meant their child could be perceived as being ‘abnormal’, whereas others felt this was not the case:

Does it mean my child's a bit abnormal because they have to go to a specialist? Adult 13 (37, F, Regular, Ext/Fill, Not‐Parent)

I wouldn't say my child, or say my niece and nephews, were abnormal because they had to get sent to a specialist. Adult 10 (53, F, Regular, Fill, Parent)

Despite concerns of case complexity and abnormality, adults accept the need for a child to be referred (irrespective of whether they were parents or not) and placed trust in the dentist by doing so:

Like our dentist said, “This is the right place to go. You're going with my blessing. These people will be lovely.” Adult 6 (37, M, Regular, Fill, Not‐Parent)

### 3.8 Theme 3: Importance of shared decision‐making for cFPM in children and young people

Choosing to restore a child's cFPM was often seen as the best decision. This was irrespective of previous treatment experiences, or whether they were a parent or not, with the suggestion that an extraction was seen as an irreversible option:

…for a child, it can only be a filling, because obviously that, getting extracted is the worst scenario really, you know its gone forever. Whereas if you have a filling, you can get the filling and then you can, you know, carry on and then make sure it doesn't happen again. Adult 5 (43, M, Regular, Ext, Parent)

Only one adult, who had no experience of filling or extraction, was not a parent and was the youngest, disagreed with this theory and felt that removal would be the only logical option:

…why bother with a filling in someone so young. If removal is an option, and it would prevent them from future issues, then it needs to be removed, and I would still feel like I'd be acting in their best interests. Adult 4 (20, M, Irregular, Nil, Not‐Parent)

Adults recognised that deciding how to manage cFPM should not be solely made solely by the child's parent:

I would try and explain to them what was going on…but ultimately my decision would be, you know, i‐, in, in partnership with the sort of dentist and my children… Adult 10 (53, F, Regular, Fill, Parent)

I would definitely endeavour to make sure that my child has a more active role in that decision‐making process. Adult 6 (37, M, Regular, Fill, Not‐Parent)

Exploring this in more depth highlighted the importance to adults about involving young people in the decision‐making process for cFPM. Their inclusion was felt to empower young people, and support their right to be actively involved in healthcare decisions:

[I've] always encouraged that two‐way conversation from the kids' point of view because as they get into adulthood, they need to understand that, that they need that conversation and that relationship with their dentist in order to make decisions about their dental health. Adult 9 (48, F, Regular, Ext/Fill, Parent)

Despite supporting their inclusion, in reality, parents rarely involved children in the healthcare decision process; they, however, acknowledged they should:

…it's up to him what he has done to his body. Obviously, we – I'll, I'll influence him as much as I can as I've also done but I'm not going to tell him – he needs to start making decisions doesn't he… Adult 11 (46, M, Regular, Ext/Fill, Parent)

In some instances, involving a child in the decision‐making process was an abstract concept—because either they were not parents or had very young children. This did not diminish the willingness to involve young people as participants often explained how they would do this either creating a hypothetical scenario, or, considering what they would have wanted to happen to them as a child:

…[I'd] try and think what they're going to be thinking about in the future. And they feel about it and how I would feel about it if it was me later on in the future, what I would have wanted to have done back when I was a kid… Adult 2 (25, M, Regular, Fill, Not‐Parent)

## 4 DISCUSSION

This qualitative study sought to critically explore young people's and adults' views of decision‐making to manage cFPMs. The importance of shared decision‐making for cFPM was evident from all participants. This process should involve the individual and their healthcare professional(s), who details risks and benefits, before a joint decision on care is made.^5^, ^18^ Young people wished to express and assert their autonomy, and to do so requires their opinion and inclusion in discussions.^5^ Where there is intent to include children's views and wishes in treatment and care options,^5^, ^7^ previous qualitative studies exploring children's experiences of participating in medical and dental care decisions suggested they are often marginalised in this experience.^19^ A small, but growing body of literature highlights that young people articulate the need to voice their preferences and be involved directly in decision‐making so as to understand what is going to happen to them.^19^, ^20^, ^21^ Much of the literature focuses on young people expressing the need for direct and clear communication about what is going to happen to them.^6^, ^7^, ^19^, ^20^, ^21^ For young people, health care is the last social domain in which they learn to assert their autonomy in decision‐making.^22^ It is likely that limited exposure to ill health, and health care, means that these situations and environments are unfamiliar, which could lead to this delay.^23^ It is, however, clear that children and young people want and should be involved in decisions around their own care as early as possible to aid their development.

This is, however, challenging and complex as competence and capacity will vary for each individual child.^7^ Professionals therefore need to think clearly about their role in this process. Favourable experiences are noted when trusting relationships are formed.^24^ Young people suggested being confidential, not withholding information, and engaging in small talk to show concern are all essential to gain trust.^25^ Providing age‐appropriate information could support children and young people in making competent and meaningful decisions.^26^ Adults wished to empower children and young people to be accountable for their own healthcare decisions. Professionals need to actively encourage adults to enact this process.^7^ The adult respondents' discourse, however, may have focussed on their ideas, rather than taking the role of a fictional parent's perspective and their responses, and may not reflect what they would do but rather what is acceptable to say. Navigating this stage in a child's development means professionals can use any opportunity to educate parents on how to involve their child in shared decision‐making.

For young people, making decisions is often shaped by various inter‐ and intra‐personal and contextual complexities. The importance of the patient–dentist relationship, and its impact on decision‐making, is evident in the literature.^18^, ^27^ Dental professionals hold a position of power in this relationship, underpinned by their knowledge and experience of the problem, which explains why young people value this opinion.^28^ Consistent with the literature, this research found that participants reported that peers influenced decision‐making. It has been reported that negative peer social judgements will influence a young person's treatment decisions to ensure ideal anterior aesthetics.^29^ It could be argued that young people's decision‐making is less susceptible to peer influence for cFPM as either restoring or extracting is unlikely to impact aesthetics. Learnt parental behaviours can shape young people's decision‐making but, in this context, they appear less important than peer judgements or professional opinion.^18^ A possible explanation for this might be that young people may not have felt it relevant to consider their parent's views when offered the chance to express their own, as in reality, they would not routinely be afforded the opportunity to disclose their views healthcare decisions are made. Alternatively, it could represent a generational shift in that young people are now actively encouraged to advocate their own rights,^23^ and therefore, the influence just does not exist. These findings contradict a growing body of evidence, which suggests young people primarily develop their decision‐making skills based on the values and behaviours learnt from their parents.^30^, ^31^ It remains, however, unclear as to what impact these learnt parental values and behaviours have in a healthcare decision‐making context.^31^ Ethnographic studies of real decision‐making situations in young people's care could help explore this finding, whilst additionally it be of value to help support future patients and parents in making a shared decision.

It is likely that previous experience or observation of a restoration being placed explains the strong preference young people had to restore cFPM. An alternative explanation could be that the asymptomatic nature of a cFPM, as per the definition used in this study, prompted a restorative decision as the patients had yet to experience any physical impact from these teeth. This preference for a child to have their cFPM restored as a first‐line option resonated with adults, irrespective of their parenthood status. This restorative preference was not as evident when adults were asked how they would manage their own cFPM. It is known that adults make decisions, both now and in future, by previous lived experiences of treatment.^32^ Other influencing factors such as functional impact, psychosocial impact, anxiety, aesthetic implications or long‐term considerations did not specifically feature in participants' accounts; these factors, however, could feature in any subconscious processing, and failure to mention does not mean they did not exist. When a restored cFPM was to become painful and further treatment was required, young people generally indicated that they would prefer to have this tooth extracted rather than undergoing a root canal treatment. A possible explanation might be that dental pain is something young people want to avoid, as it is unpleasant, and removal will almost guarantee no future pain from that tooth. Alternatively, young people may have a poor understanding of these alternative options when cFPM becomes painful. Cross‐sectional studies investigating how clinicians decide how to manage cFPM^3^ and evidence to support their clinical effectivness^33^ exist, but no studies have investigated children's decision‐making. This could establish how aware children are of the possible options for managing such clinical situations.

The phrase ‘societal norms’ refers to ideas and/or concepts that are created and accepted by the people living within a society. The term ‘filling’ may be considered a conservative social norm, as it can be identified as a less challenging procedure, meaning choosing it again is often a simpler decision as it does not require any new discussions.^34^ In contrast, extractions were considered more procedurally difficult and were classed as a non‐conservative social norm.^34^ Extractions, however, are not necessarily harder for a dentist to carry out than a filling. As such, there is a risk that decisions are being based on accepted social norms, and the perceived procedural issues, rather than patients' own personal beliefs, personal experiences or clinical presentation. Patients should be informed of all available options, tailored to their case, to permit a full discussion and minimise the potential misuse of social constructs.^34^

The possibility of a referred child being labelled as ‘abnormal’ was an interesting finding. There is scarce literature to support the concept of abnormality in health, let alone dentistry, and this merits further investigation. Case complexity extends beyond clinical needs, with socio‐economic, cultural, behavioural and environmental factors contributing to this notion.^35^ As referrals are usually made for more challenging cases,^18^ then these external factors may provide a tenuous rationale as to why some adults felt the child was abnormal.

A key strength of this study was the diverse sample, which included participants with and without experiences of cFPM. Concepts could therefore be discussed both in the reality and in the hypothetical abstract, thus understanding both stated and revealed notions and ideas. The sample did have limitations, as younger children (<12 years old) were not included, and the sample was only from England. Similarly, the views of the adults were non‐related to the young people interviewed. These patient characteristics, as previously discussed, specifically chosen as the early qualitative findings informed the design of a concurrent young person and public preference elicitation study.^10^, ^11^ Future research should include children from age 6 upwards to help capture those views and opinions of the undergoing the actual decision‐making process, rather than basing it on prior experiences.

Interviewing online allowed participants to be reached, irrespective of their circumstances or geography, compared with face‐to‐face interviews. This may, however, have unintentionally excluded those who had no access to technology, a poor internet connection or those who did not feel competent in using technology.

A key strength was speaking directly to young people about their views, rather than using their parents as proxy. Undertaking an interview may be complex and challenging for young people, and having a parent/guardian present may help; having a parent present, however, might result in the young person being told what to say, whether they have an opinion or not.^36^, ^37^ In this study, it was made clear to the two parents who supported their child's interview that we wanted to hear their child's opinions. Field notes of non‐verbal interactions and re‐reading the manuscripts highlighted that in both cases, the parents did very little to influence responses with both parents either re‐phrasing challenges questions or encouraging participation when nearing the end of the interview.

Developing and using Figure 1 was a methodological strength, ensuring the interviews remained focussed on just the cFPM and was less intrusive than asking participants to identify their own cFPM.

In conclusion, several factors influence how decisions about cFPM are made by adults and young people. The gap in perceived shared decision‐making and actively involving young people was apparent. This study strengthens the evidence around supporting decision‐making for cFPM.

## AUTHOR CONTRIBUTIONS

GDT contributed to the overall design of the study; development of topic guides; undertaking and analysing interviews; discussing the data; and compiling the manuscript. CE, NI and CRV contributed to the overall design of the study; discussing the data; and reviewing and refining the manuscript.

## CONFLICT OF INTEREST STATEMENT

Dr. Greig Taylor was funded by an NIHR Doctoral Research Fellowship (NIHR300251). The views expressed are those of the author(s) and not necessarily those of the NIHR or the Department of Health and Social Care.

## ETHICS STATEMENT

This study received a favourable ethical opinion from the North of Scotland NHS ethics service (20/NS/0124; 22 October 2020). Appropriate informed consent and assent of participants were obtained.

## Supporting information


Appendix S1.

## Data Availability

The data that support the findings of this study are available from the corresponding author upon reasonable request.

## References

[1] Ismail AI , Sohn W , Tellez M , et al. The International Caries Detection and Assessment System (ICDAS): an integrated system for measuring dental caries. Community Dent Oral Epidemiol. 2007;35(3):170‐178. 17518963 10.1111/j.1600-0528.2007.00347.x

[2] Lygidakis NA , Garot E , Somani C , Taylor GD , Rouas P , Wong FSL . Best clinical practice guidance for clinicians dealing with children presenting with molar‐incisor‐hypomineralisation (MIH): an updated European Academy of Paediatric Dentistry policy document. Eur Arch Paediatr Dent. 2022;23(1):3‐21. 34669177 10.1007/s40368-021-00668-5 PMC8926988

[3] Taylor GD , Pearce KF , Vernazza CR . Management of compromised first permanent molars in children: cross‐sectional analysis of attitudes of UK general dental practitioners and specialists in paediatric dentistry. Int J Paediatr Dent. 2019;29(3):267‐280. 30657228 10.1111/ipd.12469

[4] Somani C , Taylor GD , Garot E , Rouas P , Lygidakis NA , Wong FSL . An update of treatment modalities in children and adolescents with teeth affected by molar incisor hypomineralisation (MIH): a systematic review. Eur Arch Paediatr Dent. 2022;23(1):39‐64. 34110615 10.1007/s40368-021-00635-0 PMC8927013

[5] NICE . NICE guideline [NG197]—Shared decision making [Internet]. 2021. Accessed June 7, 2022. https://www.nice.org.uk/guidance/ng197/chapter/Recommendations#embedding‐shared‐decision‐making‐at‐an‐organisational‐level

[6] Grootens‐Wiegers P , Hein IM , van den Broek JM , de Vries MC . Medical decision‐making in children and adolescents: developmental and neuroscientific aspects. BMC Pediatr. 2017;17(1):120. 28482854 10.1186/s12887-017-0869-x PMC5422908

[7] Coyne I , Harder M . Children's participation in decision‐making: balancing protection with shared decision‐making using a situational perspective. J Child Health Care. 2011;15(4):312‐319. 21828162 10.1177/1367493511406570

[8] United Nations . Convention on the rights of the child. 1989.

[9] Tong A , Sainsbury P , Craig J . Consolidated criteria for reporting qualitative research (COREQ): a 32‐item checklist for interviews and focus groups. International J Qual Health Care. 2007;19(6):349‐357. 10.1093/intqhc/mzm042 17872937

[10] NIHR Funding and Awards . Dental ExtraCtion versus filling of adult teeth In chilDren: A cost Effectiveness analysis (DECIDE) [Internet]. Accessed September 12, 2023. https://fundingawards.nihr.ac.uk/award/NIHR300251

[11] Coast J , Al‐Janabi H , Sutton EJ , et al. Using qualitative methods for attribute development for discrete choice experiments: issues and recommendations. Health Econ. 2012;21(6):730‐741. 21557381 10.1002/hec.1739

[12] Saunders B , Sim J , Kingstone T , et al. Saturation in qualitative research: exploring its conceptualization and operationalization. Qual Quant. 2018;52(4):1893‐1907. 29937585 10.1007/s11135-017-0574-8 PMC5993836

[13] Geddis‐Regan AR , Exley C , Taylor GD . Navigating the dual role of clinician‐researcher in qualitative dental research. JDR Clin Transl Res. 2022;7(2):215‐217. 10.1177/2380084421998613 33618559

[14] Glaser BG . The constant comparative method of qualitative analysis. Soc Probl. 1965;12(4):436‐445.

[15] QSR International . NVivo version 1.7. QSR International; 2021.

[16] Braun V , Clarke V . Using thematic analysis in psychology. Qual Res Psychol. 2006;3(1):77‐101.

[17] Elliott V . The research interview: reflective practice and reflexivity in research processes. Int J Res Method Educ. 2018;41(2):237‐238.

[18] Osborne R , Silva M , Taylor GD . Qualitative study exploring general dental practitioners’ views of MIH and its management in the UK and Australia. Int J Paediatr Dent. 2023. doi:10.1111/ipd.13135 37969051

[19] Coyne I , Amory A , Kiernan G , Gibson F . Children's participation in shared decision‐making: children, adolescents, parents and healthcare professionals' perspectives and experiences. Eur J Oncol Nurs. 2014;18(3):273‐280. 24629505 10.1016/j.ejon.2014.01.006

[20] Jenkins L , Hepburn A , MacDougall C . How and why children instigate talk in pediatric allergy consultations: a conversation analytic account. Soc Sci Med. 2020;266:113291. 32920197 10.1016/j.socscimed.2020.113291

[21] Wang YW , Carnevale FA , Ezcurra M , et al. The moral experiences of children with osteogenesis imperfecta. Nurs Ethics. 2022;29(7–8):1773‐1791. 35801828 10.1177/09697330221105635 PMC9667074

[22] Smetana JG , Campione‐Barr N , Daddis C . Longitudinal development of family decision making: defining healthy behavioral autonomy for middle‐class African American adolescents. Child Dev. 2004;75(5):1418‐1434. 15369523 10.1111/j.1467-8624.2004.00749.x

[23] Wray‐Lake L , Crouter AC , McHale SM . Developmental patterns in decision‐making autonomy across middle childhood and adolescence: European American parents' perspectives. Child Dev. 2010;81(2):636‐651. 20438465 10.1111/j.1467-8624.2009.01420.x PMC2864944

[24] Davison G , Kelly MA , Conn R , Thompson A , Dornan T . How do children and adolescents experience healthcare professionals? Scoping review and interpretive synthesis. BMJ Open. 2021;11(7):E054368. 10.1136/bmjopen-2021-054368 PMC8273482 34244289

[25] Klostermann BK , Slap GB , Nebrig DM , Tivorsak TL , Britto MT . Earning trust and losing it: adolescents' views on trusting physicians. J Fam Pract. 2005;54(8):679‐687. 16061053

[26] Mårtenson EK , Fägerskiöld AM . A review of children's decision‐making competence in health care. J Clin Nurs. 2008;17(23):3131‐3141. 18005126 10.1111/j.1365-2702.2006.01920.x

[27] Muirhead VE , Marcenes W , Wright D . Do health provider–patient relationships matter? Exploring dentist‐patient relationships and oral health‐related quality of life in older people. Age Ageing. 2014;43(3):399‐405. 24275429 10.1093/ageing/aft183

[28] Isaacs D . Trust me I'm a doctor: making decisions about children's direction of care. J Paediatr Child Health. 2019;55(12):1411‐1413. 31846156 10.1111/jpc.14588

[29] Henson ST , Lindauer SJ , Gardner WG , Shroff B , Tufekci E , Best AM . Influence of dental esthetics on social perceptions of adolescents judged by peers. Am J Orthod Dentofacial Orthop. 2011;140(3):389‐395. 21889084 10.1016/j.ajodo.2010.07.026

[30] Donnelly M , Kilkelly U . Participation in healthcare: the views and experiences of children and young people. Int J Child Rights. 2011;19(1):107‐125.

[31] Maitre L , Julvez J , López‐Vicente M , et al. Early‐life environmental exposure determinants of child behavior in Europe: a longitudinal, population‐based study. Environ Int. 2021;153(1):106523. 33773142 10.1016/j.envint.2021.106523 PMC8140407

[32] Vernazza CR , Steele JG , Whitworth JM , Wildman JR , Donaldson C . Factors affecting direction and strength of patient preferences for treatment of molar teeth with nonvital pulps. Int Endod J. 2015;48(12):1137‐1146. 25400281 10.1111/iej.12413

[33] Taylor GD , Vernazza CR , Abdulmohsen B . Success of endodontic management of compromised first permanent molars in children: a systematic review. Int J Paediatr Dent. 2020;30(3):370‐380. 31778237 10.1111/ipd.12599

[34] Brabers AEM , van Dijk L , Groenewegen PP , de Jong JD . Do social norms play a role in explaining involvement in medical decision‐making? Eur J Public Health. 2016;26(6):901‐905. 27161909 10.1093/eurpub/ckw069

[35] Safford MM , Allison JJ , Kiefe CI . Patient complexity: more than comorbidity. The vector model of complexity. J Gen Intern Med. 2007;22(3):382‐390. 10.1007/s11606-007-0307-0 PMC2219701 18026806

[36] Einarsdóttir J . Research with children: methodological and ethical challenges. Eur Early Child Educ Res J. 2007;15(2):197‐211.

[37] Ponizovsky‐Bergelson Y , Dayan Y , Wahle N , Roer‐Strier D . A qualitative interview with young children: what encourages or inhibits young children's participation? Int J Qual Methods. 2019;18(1):1.

