# Supplementary material for: Young people's and adults' views and experiences of decision‐making to manage compromised first permanent molars: a qualitative study
Source: Int J Paediatr Dent. 2024 May 27;35(1):165–75. doi: 10.1111/ipd.13217 (PMC11626489; doi:10.1111/ipd.13217)
Supplement: Supplementary file 1 — Appendix S1. [file IPD-35-165-s001.docx]

**DECIDE Study**

**WP2 - Qualitative Interviews**

**Topic Guide**

**This interview will be recorded. Anything you say will be anonymised. Can you re-confirm you have read the Participant Information Sheet and consent to this interview?**

**START RECORDING**

**SECTION 1**

**Attendance**

- Frequency of attendance?
- Reasons for attendance?
  - (Prompt: pain, values oral health, trust dentist’s opinion)
- Attendance important for you? Family member? Child?
- Attendance other than dentist for dental care?
  - (Prompt: Why, where?)

**Experiences**

- Previous dental experience
  - (Prompt: types of treatments; Positive/negative experiences)
- Important to have proposed treatment fully explained for overall experience
- Tell me about a time when you’ve had to have treatment done?
  - (Prompt: What happened? Length? Painful? Emotional? Relief? Short- and long-term effects?)
- **Expectations**
- What do you expect to obtain in attending your dentist?
- What do you expect your dentist to provide you?
  - (Prompt: A child, or you child?)

**PAGE 1**

**SECTION 2**

**Identifying the 1^st^ Permanent Molar**

- Are you aware that humans have two sets of teeth?

*(Share a picture of a mouth with all teeth present – number or colours to help identify on zoom)*

- Which colour represents the molar teeth?
- Which letter is the **first permanent molar**?
- Can you recall if you have ever required treatment on your **first permanent molar**?
  - (Prompt : If definitive yes, continue with experiences; if no, go to haven’t had treatment)

**Experiences with these teeth**

- Problems with this **first permanent molar**?
  - (Prompt: pain, can’t chew, can’t sleep, etc)
- Dental disease on this **first permanent molar**?
  - (Prompt: Dentist ever mentioned specific disease? How did you feel knowing disease on this tooth? Worried? Future problems?)
- Treatment for this **first permanent molar**?
  - (Prompt: what treatment? Explain what happened? How did it feel? More than one episode of care? Still have the tooth?)

**PAGE 2**

**THOSE WHO HAVE HAD TREATMENT**

- Have you heard of treatments fillings and extractions? Do you know what they mean? What does mean to me?
- How did it feel having a filling and/or extraction of this **first permanent molar**?
  - (Prompt: painful during? After? Sensations? Worries?)
  - Are fillings “normalised”?
- Why that treatment over the alternative (e.g filling over extraction) for **first permanent molar**?
  - (Prompt: avoidance of pain? Previous experiences? Peer influence? Type or colour of the filling material? Impact on eating/chewing? Costs? Long term maintenance needs? Size of defect
  - **(Additional adolescent prompts: parental influence? Dental influence)**
- How did you decide to fill or extract for the **first permanent mola**r?
  - (Prompt: help of dentist? Who did treatment help decide? Important for you to have your dentist do treatment? Seeking specialist care differ? Severity of defect? Expectation that treatment will work help decide?)
  - **(Additional adolescent prompts: help of parent?)**
- How much of a priority, to you, was keeping the **first permanent molar**?
  - (Prompt: If became sore after treatment, would you still want to keep it? What would change your mind? Would having a gap matter at the back? Different if at the front? Leave gap, or place false tooth – why?)
  - **(Additional adolescent prompts: felt it was important to your parent to keep tooth?)**
- Decision change between acquired and developmental conditions for compromised **first permanent molar**?

**PAGE 3**

**THOSE WHO HAVEN’T HAD TREATMENT**

**(Imagine you visit dentist at they tell you that you have a dental problem with this first molar tooth, and you need something done)**

- Have you heard of treatments fillings and extractions? Do you know what they mean?
- How would you feel if you had to have a filling of this **first permanent molar**? Extraction of this **first permanent molar**?
  - (Prompt: painful during? After? Sensations? Worries? Different if extraction?)
- Happy with Injection only?
  - (Prompt: why? Prefer Alternatives offered if treatment were to be done again – sedation, GA)
- What factors would help make that decision to either have your **first permanent molar** filled or removed?
  - (Prompt: Avoidance of pain? Severity/size of the defect? Previous experiences on other teeth? Peer influence? Dentist opinions? Type or colour of the filling material? Impact on eating/chewing? Costs? Long term maintenance needs? Clear information? Expectation that treatment will work help decide )
  - **(Additional adolescent prompts: parent help? Would parents influence your decision? Dentist vs. adult opinion? Have you been involved in decision making?)**
- Importance of your dentist doing treatment.
  - (Prompt: what about seeing a specialist?)
- How much of a priority, to you, was keeping that **first permanent molar**?
  - (Prompt: If became sore after filling, would you still want to keep it? What would change your mind to extraction? Would having a gap matter at the back? Different if at the front? Leave gap, or place false tooth – why?)
  - **(Additional adolescent prompts: felt it was important to your parent to keep tooth? Parental experiences of false teeth? Parental influence on when to lose tooth?)**
- Decision change between acquired and developmental conditions for compromised **first permanent molar**?

**PAGE 4**

**Section 3**

**‘Hypothetical child’ (FOR ADULTS ONLY)**

- Do you have any children?
- If so, what how many and what are their ages?
- How would you feel if your child had to have a filling of their **first permanent molar**?
  - (Prompt: painful during? After? Sensations? Worries? Different if extraction?)
- Would you be happy for your child to have an injection only?
  - (Prompt: why? Prefer Alternatives offered if treatment were to be done again – sedation, GA)
- What factors would help make that decision to either have your child’s **first permanent molar** filled or removed?
  - (Prompt: How would your child feel? Blame parents? Social norms - how would their friends think of them? Severity of defect? Avoidance of pain? Previous experiences on other teeth? Peer influence? Dentist opinions? Type or colour of the filling material? Impact on eating/chewing? Costs? Long term maintenance needs?
- How important to you is it that your dentist does the treatment for your child’s **first permanent molar?**
  - (Prompt: what about seeing a specialist?)
- How much of a priority is keeping your child’s **first permanent molar**?
  - (Prompt: If became sore after filling, would you still want to them to keep it? Would mind them having a gap at the back? Different if at the front? Would you want them to have this gap left, filled or closed with braces?– why?)
- Involvement of children in decision making? When you were a child, were you involve in decision making?
- **Is there anything else you would like to discuss, or feel that we haven’t**

**covered?**

- **End of Recording**

**PAGE 5**
